# Effects of Emotional States on the Spatial Perception of Youth Athletes with Different Alerting Efficiencies

**DOI:** 10.5114/jhk/187257

**Published:** 2024-07-17

**Authors:** Lian Wang, Qiao Meng, Mariusz Lipowski

**Affiliations:** 1 Department of Physical Education, Chengdu Sport University, Chengdu, China.; 2 Faculty of Physical Culture, Gdansk University of Physical Education and Sport, Gdańsk, Poland.; 3 Faculty of Social and Humanities, WSB Merito University Gdansk, Gdańsk, Poland.

**Keywords:** spatial perception, sport, attention, alerting, emotion, youth athletes

## Abstract

The purpose of this study was to explore the role of the attentional alerting network in spatial perceptual processing in youth athletes under different emotional states. Two hundred and fifty participants were recruited to assess alerting efficiency using the Attention Network Test and were divided into high and low alerting efficiency groups based on the front and back 27% of the alerting scores as a dividing metric. Subsequently, participants completed the Spatial Perception Response Task under different emotionally induced conditions. Results showed a correlation between alerting efficiency and spatial perception, the higher the alerting efficiency, the faster and more accurate the spatial perception response. Emotion and spatial location interacted significantly (p < 0.05), and subjects in the angry emotional state had faster spatial responses to the lower left visual field than those in the happy emotional state. Moreover, an interaction was found for location accuracy (p < 0.05). The high alerting efficiency group showed lower accuracy in the anger state than in the happy state. These findings suggest that alerting efficiency can be used as an objective indicator for assessing the spatial perceptual ability of youth athletes, which provides empirical evidence for mental selection and perceptual training of youth athletes.

## Introduction

In the field of sports, perceptual functions play a critical role in executing tasks and movements in various competitions, and athletes’ perception has been extensively studied in sports psychology (Barrett et al., 2020; Notarnicola et al., 2014). Spatial perception is the process by which an individual perceives specific shapes, spatial orientations, sizes, distances, and other characteristics of objects (Witt, 2020). Among the specialized perception abilities of athletes, spatial perception is a crucial component, as athletes' spatial orientation and judgment of their surroundings are essential. During sports competitions, athletes need to perceive a variety of spatial relationships that are relatively stationary (e.g., penalty kicks and free throws), yet more often spatial relationships that are relatively in motion (e.g., actions such as serving and swinging to hit the shuttlecock in badminton, the ball in table tennis and tennis). Visual spatial skill is a very important coordinative ability for athletes, especially ball players, whose motor responses in a variety of situations require adequate spatial assessment (Wolbers and Hegarty, 2010). This is because it involves not only the correct perception of their own and opponents' spatial characteristics and their interrelationships, but also the accurate perception of the spatial position of the object in motion ( e.g., a ball) and its relationship to oneself and opponents (Savelsbergh et al., 2002). Therefore, athletes' spatial position perception ability is an important part of their specialized perception ability and an important factor in the effectiveness of athletes' competitive performance.

Attention has been identified as a crucial factor influencing spatial perception and plays an irreplaceable role in athletes' motor performance (Moran, 2017). Attention is a complex construct and can be divided into two primary aspects: selective attention and sustained vigilant attention (Sturm and Willmes, 2001). Most previous studies on the effects of attention on spatial perception have focused on the effects of selective attention (Kirsch et al., 2021), while the role of the non-selective alerting component of attention in spatial perceptual processing has been less studied, especially in the motor domain. Alerting, as a form of sustained attention, is critical for successful athletic performance (Pei et al., 2022). According to the Posner and Petersen's (1990) attentional network model, attention consists of three separate networks: alerting, orienting, and executive control. Fan et al. (2002) developed the Attentional Network Test (ANT) which consists of a single task that simultaneously assesses the efficiency of each attentional network (Fan et al., 2002). There are two types of alerting: phasic alerting and intrinsic alerting (Sturm and Willmes, 2001). Phasic alerting is specific to a particular task, whereas intrinsic alerting refers to general cognitive arousal. In this study, we focused on phasic alerting which refers to the ability to acquire and maintain a high state of internal arousal, enabling individuals to be prepared to respond to stimuli that will arise (Raz and Buhle, 2006).

Research has shown that spatial perception is influenced not only by structural factors related to attention, but also by emotional variables (Aliberti et al., 2023; Rossi and Pourtois, 2012). Emotions are the processes by which individuals initiate, regulate or maintain the degree and timing of the production of internal affective states, and a range of related physiological processes (Eisenberg and Spinrad, 2004). Negative emotions have been found to bias spatial localization perception in individuals, even in low-load spatial localization tasks, and reduce the accuracy of spatial localization responses to peripheral stimuli (Rossi and Pourtois, 2013). In competitive contexts, athletes are inevitably influenced by emotions, and several studies have demonstrated the important role emotions play in athletes' performance and competitive outcomes.

It is well established that emotions can impact various attentional processes, including concentration, distraction elimination, and attentional transfer (Compton et al., 2004). However, previous research has tended to view attention as a single cognitive process and attentional functioning because of the holistic tests, whereas in fact, attention encompasses different attentional networks (Dennis et al., 2008). Most current research has used ANT tasks to assess the effects of emotions on attention. For example, one study used a modified version of the ANT paradigm to examine how threat-related attentional biases affected the attentional system and found that anxiety significantly moderated the functioning of the attentional network, where anxiety positively correlated with alerting, whereas fear and neutral faces were correlated with a decrease in alerting efficiency (Dennis et al., 2008). However, in a study examining the effects of task-irrelevant emotional stimuli on attentional networks, it was discovered that for simple tasks, threat (fear and anger) facilitated the orienting network relative to happy emotional stimuli, but did not have a significant effect on the alerting network (O’Toole et al., 2011). Contrary to this, the results of the study showed that subjects in the emotion of anger reacted faster than the control group (response time), showing a greater alerting effect, whereas the effect of anger on the orienting and executive control networks was not significant (Techer et al., 2015). These inconsistent effects of emotion on the attentional network found in the abovementioned studies may be related to the type of emotion, task difficulty, and stimulus duration. Summarizing the current research, there is scarcity of studies investigating the role of the attentional alerting network in spatial perceptual processing. Additionally, the effects of emotional stimuli on the alerting network exhibit further inconsistency, with conflicting findings regarding whether they increase or decrease its activity. Research has shown that emotions affect spatial perception. Rossi and Pourtois (2013) found in an experimental study that spatial perception depended not only on structural factors in terms of attention, but also on emotional variables. Furthermore, it was found that angry emotional states might produce early perceptual biases, such as spatial localisation perception (Pourtois et al., 2013). Even in a low load spatial localization task, negative emotions reduced the accuracy of spatial localisation responses to peripheral stimuli (Eisenberg and Spinrad, 2004). The above studies suggest that emotion is an important factor affecting spatial perception, and negative emotions can lead to a decline in spatial perceptual ability.

Therefore, this study explored the effects of different emotional states on the spatial perceptual responses of youth athletes with different alerting efficiency through an experimental approach (Attention Network Test (ANT) and a spatial perceptual response task experiment). The aim of this study was to investigate the factors that influenced spatial perception in youth athletes and to identify their patterns of variation. Such findings could inform the psychological selection and spatial perception training of youth athletes, providing a scientific reference and empirical basis for this field.

In summary, we hypothesized that alerting efficiency could have a positive effect on the spatial perception of youth athletes and that there might be an interaction effect between emotional state and alerting efficiency on their spatial perception.

## Methods

### Participants

We used the automatic direct method available in G*Power (version 3.1.9.7) to estimate the necessary sample size, with a medium effect size of 0.25, the level of significance at α = 0.05, power = 0.95. The sample size was calculated to include at least 52 individuals. Two hundred and fifty well trained youth athletes (98 females; age = 16.36 ± 1.26 years; 152 males; age = 15.59 ± 1.12 years) were randomly recruited through advertisements posted in a sports university and its affiliated sports schools in China. The type of selected sports included mainly round-based ball games (i.e., badminton, table tennis and tennis). Participants all met the following criteria: (1) three or more years of professional training experience; (2) qualified as a national player at the second grade or above; (3) more than three training sessions per week in the past two years; (4) more than two hours of training time per session; (5) right-handed, with reported normal or corrected naked eye vision, and with no color weakness or color blindness; (6) no reported diagnosed psychiatric or neurological disorders, not taking any medication that would influence central nervous system functioning, and (7) lack of previous experience in ANT testing. Written informed consent was given in accordance with the procedures and protocols approved by the ethics committee of the of the Chengdu Sport University (protocol code: 20212801H; approval date: 28 September 2021).

### Stimuli and Tasks

#### Attention Network Test (ANT)

The Attention Network Test (ANT) was presented using E-Prime 2.0 software (Psychological Software Tools, Pittsburgh, PA, USA) to display the stimuli on a computer screen with a 19-inch monitor (screen resolution of 1024 x 768 pixels, refresh rate of 85 Hz). Participants’ eyes were approximately 60 cm away from the computer screen. The standard procedures for the ANT were followed (https://www.sacklerinstitute.org/cornell/assays_and_tools/ant/jin.fan/; accessed on 22 October 2021).

**Figure 1:**
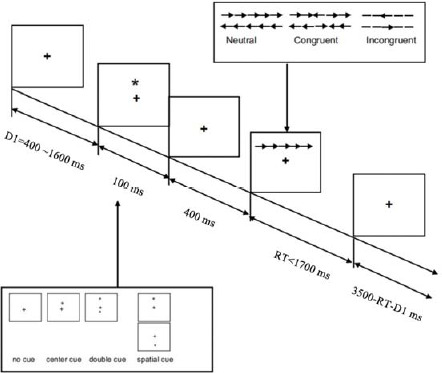
Schematic overview of the Attention Network Test adopted in this study. Notes: The symbol “*” indicates the cue, and the symbol “+” represents the fixation in the center of the screen.

The ANT contained three experimental blocks with 96 trials each, and each trial comprised five events: (a) first, a "+" gaze point was presented in the center of the screen for 400–1600 ms; (b) a cue "*" signal was presented for 100 ms; (c) a gaze point was presented at the center of the screen for 400 ms; (d) afterwards, the target stimulus requiring a response was presented for no more than 1700 ms and it disappeared immediately once the participant pressed a key. However, the target gaze point kept showing in a variable of time. The duration of the target gaze point after the response was 3500 ms minus the duration of the first gaze plus the response time; (e) the next trial was then started, and the total duration of each trial procedure was 4000 ms (Figure 1).

The ANT can effectively distinguish among network functions such as alerting, orienting, and executive control, and use the difference between reaction times under different cue conditions to represent different attentional network functions. Alerting efficiency = RT_no cue_ − RT_double cue_; Orienting efficiency = RT_center cue_ − RT_spatial cue_; Executive Control efficiency = RT_incongruent_ − RT_congruent_ (Fan et al., 2005).

#### Spatial Perceptual Selection Response Tasks

**Figure 2:**
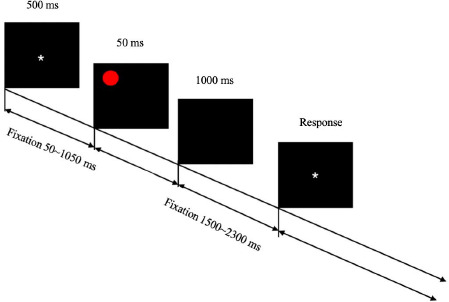
Schematic diagram of the spatial perception reaction task. Notes: The symbol “*” represents the fixation in the center of the screen, and the red spherical pattern represents visual stimulation

Spatial orientation perception is an important indicator of an athlete's ability to master technical movements (Farrow and Reid, 2012). Therefore, in this study, a spatial perception selection response task was used to investigate youth athletes' spatial orientation perception. The experimental setup was as follows: a red spherical pattern with a diameter of 5.73° visual angle was presented as a visual stimulus on a 19-inch computer screen at different positions in the visual field, located at the upper left, lower left, upper right, and lower right with a black background. The experiment consisted of a preparation phase and a formal experiment. In the preparation phase, the participant pressed any key to start the experiment and was promptly provided with the following instructions: "Please respond with the corresponding key to the position of the red spherical stimulus on the screen. Press 1 for the upper left, 2 for the upper right, 4 for the lower left, and 5 for the lower right. Respond quickly and accurately. Press any key to start the experiment when ready." After the participant pressed any key, a white "*" fixation point appeared in the center of the black screen for 500 ms. The time interval between the fixation points and the spherical stimulus ranged from 50 to 1050 ms. Then, a random red spherical stimulus was presented for 50 ms. After that, a black screen was presented for 1000 ms, and the participant was required to respond based on the position of the red spherical stimulus on the black screen. The time interval between one response and the next stimulus was randomized, ranging from 1500 to 2300 ms. Each experiment included four sets, with 25 stimuli in each set, for a total of 100 stimuli (Figure 2).

#### Emotion Inducing Stimuli

Viewing videos has been found to be a simple and effective way to induce emotions in research, which is easy to control and has good effects (Porzig-Drummond et al., 2009). The happy emotion video was selected from the “Family Humour Video" and lasted for 4 min and 50 s; the angry emotion video clip was selected from the "Anti-Child Abuse Advertisement" and lasted for 4 min and 45 s. To ensure the equivalence between the two emotional induction materials, the selected videos were subjectively evaluated by 30 youth athletes using a five-point Likert scale (1 = not at all, 5 = extremely). The average reported level of happiness for the happy emotion video was 3.69, and the average reported level of anger for the angry emotion video was 3.72. One-sample *t*-tests were performed and the result of 3 was obtained as the median of the average degree of emotionally induced; these results showed significant differences (*p* < 0.05), indicating that the selected videos in the experiment were effective in inducing emotions.

#### Emotion Assessment Scale

The Emotion Assessment Scale is a self-administered scale drawing on previous research (Todd et al., 2015). Participants were asked to select the score that best expressed their level of feeling after two emotional words (0 = not at all ~8 = very strongly). Two words represented anger, and two words represented happiness, with higher numbers indicating the strength of the participant’s emotion. Such self-report measures of emotional experience are often used to assess the effectiveness of emotion manipulation in studies of this type (Fredrickson and Branigan, 2005).

#### Design and Procedures

All participants first completed the ANT which identified high and low subgroups based on the pre and post 27% of the alertness network scores as a dividing metric, and 135 volunteered to participate in the follow-up experiment. They were randomly allocated to either the happy or the angry emotion group, and their emotions were induced through video. Subsequently, they completed the Spatial Perceptual Selection Response Task, which lasted eight minutes. Finally, they filled out the Emotional Assessment Scale and underwent an emotion manipulation test to confirm successful emotion elicitation. For the angry emotion group, appropriate post experiment treatment was provided. All participants received a certain amount of compensation upon completion of the experiment.

Additionally, as this study aimed to examine the impact of specific emotions on experimental variables, participants who indicated "not at all" on the target emotion dimension of the emotion manipulation test were excluded from the analysis. A total of 135 participants took part in this experiment. Following the exclusion of invalid data, a total of 130 valid datasets were obtained (48 females), and participants were randomly assigned to one of the four groups as follows: 1) a high alerting efficiency-happy group with 30 participants; 2) a high alerting efficiency-angry group with 33 participants; 3) a low alerting efficiency-happy group with 34 participants; 4) a low alerting efficiency-angry group with 33 participants. A significant difference in alerting efficiency was found between the high (64.46 ± 11.66) and the low alerting efficiency group (24.30 ± 9.33), (t (128) = 15.79, *p* < 0.001, *d* = 1.18), indicating that the experimental groupings were reasonable.

Individual performance was evaluated in terms of the mean response time for spatial selection (RT) and the mean percentage of the correct spatial location judgment (accuracy). RT was defined as the time between the appearance of the red spherical stimulus and pressing the corresponding button. The response was considered correct based on the different visual field positions of the red ball landing on the screen.

### Statistical Analysis

Prior to factor analysis, normality tests were performed on the spatial response times and response accuracy of the different alerting groups. The skewness ranged between −0.13 and 0.48 and the kurtosis ranged from −1.12 to 0.84. Hence the distribution of the data was assumed as normal. The statistical analyses were carried out using the software package SPSS version 25. The level of significance of α = 0.05 was assumed for all statistical procedures.

We applied a two (alerting) × two (emotion) × four (spatial location) mixed-factor design analysis of variance (ANOVA) separately on accuracy and RTs. Alerting, which included high alerting efficiency and low alerting efficiency (2), and emotion, which contained angry and happy emotions (2), were intersubjective factors. The spatial location was a within-subject factor which consisted of four sub-factors, i.e., top left, bottom left, top right, and bottom right (4). The dependent variables used in the experiment included RTs and Accuracy.

## Results

### Emotion Manipulation Check

To test the validity of emotion elicitation, the mean scores of two similar words were used to represent the happy and angry emotions. An independent sample *t*-test revealed that the happy group reported significantly higher happy rating scores than the angry group (*M* = 5.10, *SD* = 1.25, *t* (128) = 22.08, *p* < 0.001, *d* =1.19) and the angry group reported significantly higher anger rating scores than the happy group (*M* = 5.11, *SD* = 1.53, *t* (128) = 18.50, *p* < 0.001, *d* = 0.96). The results suggest that emotion elicitation was successful; *p*-value was considered significant when it was less than 0.05.

### Behavioural Results

**Figure 3:**
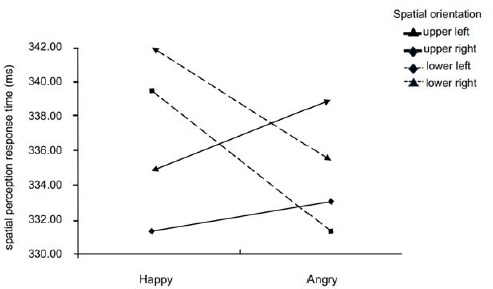
Interaction Emotion × Space Location on response time.

The study used the Greenhouse-Geisser degrees of freedom correction method for repeated measures ANOVA. RTs showed a main effect of alerting [*F* (1,126) = 9.48, *p* = 0.003, *η^2^p* = 0.20], with shorter RTs in the high alerting efficiency group than in the low alerting efficiency group (331 ms [328–335 ms] vs. 339 ms [335–342 ms]). Moreover, an Emotion × Space Location interaction [*F* (3,378) = 2.78, *p* = 0.041, *η^2^p* = 0.08] was found for RTs. Further simple effects analysis revealed no significant differences between youth athletes' spatial perceptual response times in different emotional states, the only difference was that in the lower left visual field, athletes reacted more quickly to spatial perception in the angry emotional state than in the happy emotional state (329 ms [324–334 ms] vs. 340 ms [333–345 ms] (Figure 3). No significant effects were found for the space location, nor for interactions (Table 1).

**Table 1:** Main and interaction effects of mixed-measure ANOVA on response time.

Response Times	df	*F*	*p*	*η* ^2^ *p*	Effect Size
Space Location	3,378	2.06	0.105	0.02	Small
Alerting	1,126	9.48**	0.003	0.20	Large
Emotion	1,126	5.20*	0.029	0.13	Medium
Alerting × Space Location	3,378	0.67	0.580	0.01	Small
Alerting × Emotion	1,126	1.28	0.260	0.01	Small
Space Location × Emotion	3,378	2.78*	0.041	0.08	Medium
Alerting × Emotion × Space Location	3,378	0.97	0.409	0.01	Small

Notes: * p < 0.05, ** p < 0.01

Mixed factor ANOVA of accuracy showed a main effect of the space location [*F*(3,378) = 5.62, *p* = 0.001, *η^2^p* = 0.14], Post-hoc tests revealed that accuracy in the bottom left (mean ([95% confidence interval]: 98.7% [98.5–98.9%]) was lower than in the top left (mean ([95% confidence interval]: 99.3% [99.1–99.4%]) and the top right (mean ([95% confidence interval]: 99.1% [98.9–99.3%]) (*p* < 0.01). Moreover, a main effect of alerting [*F* (1,126) = 10.15, *p* = 0.002, *η^2^p* = 0.11] was found with the high alerting efficiency group showing higher accuracy than the low alerting efficiency group (99.2% [99.0–99.3%] vs. 98.9% [98.8–99.0%].

**Figure 4:**
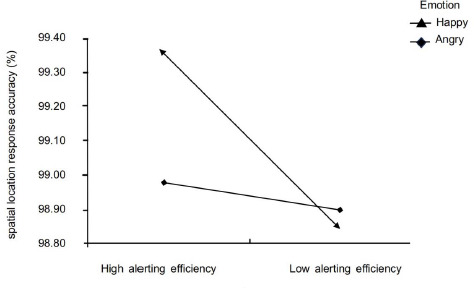
Interaction Emotion × Alerting on response accuracy.

Furthermore, an interaction was found for accuracy [*F* (1,126) = 6.22, *p* = 0.014, *η^2^p* = 0.09]. The high alerting efficiency group showed lower accuracy in the anger state than in the happy state (99.0% [98.8–99.2%] vs. 99.4% [99.2–99.5%]; and in the happy state, the accuracy of the spatial location judgment was higher in the high alerting efficiency group than in the low alerting efficiency group (99.4% [99.2–99.5%] vs. 98.9% [98.7–99.0%] (Figure 4). No significant effects were found for the space location, nor for interactions (Table 2).

**Table 2:** Main and interaction effects of mixed-measure ANOVA on response accuracy.

Accuracy	df	*F*	*p*	*η* ^2^ *p*	Effect Size
Space Location	3,378	5.62**	0.001	0.14	Medium
Alerting	1,126	10.15*	0.002	0.11	Medium
Emotion	1,126	3.11	0.080	0.02	Small
Alerting × Space Location	3,378	1.90	0.129	0.02	Small
Alerting × Emotion	1,126	6.22*	0.014	0.09	Medium
Space Location × Emotion	3,378	1.31	0.270	0.01	Small
Alerting × Emotion × Space Location	3,378	1.73	0.161	0.01	Small

Notes: * p < 0.05, ** p < 0.01

## Discussion

This study adopted the Attention Network Test (ANT) and a spatial perceptual response task to investigate the effects of emotion on spatial perception in youth athletes with different alerting efficiencies. Results showed a significant main effect of alerting efficiency in the experiment, indicating that higher alerting efficiency was associated with faster and more accurate spatial perceptual choice responses. It has been shown that alerting can be widely distinguished as intrinsic (non-phasic) and extrinsic (phasic) alerting (Sturm and Willmes, 2001). Intrinsic alerting refers to the internal (top-down) control of the level of the organism’s arousal that can be observed during a simple reaction time (RT) task, without a previous alerting stimulus. In contrast, extrinsic alerting refers to a relatively short-lived change that occurs in an individual following the perception of external cues or alerting stimuli during the preparatory process of the organism's cognitive system, which is a "bottom-up" perceptual process (Petersen and Posner, 2012). Extrinsic alerting can be enhanced by stimuli such as alerting signals (Raz and Buhle, 2006), and alerting signals periodically increase an individual's arousal level. In this experiment, in which the spatial perceptual response task was selected, a gaze point was presented before the target stimulus appeared in each trial, and the time interval between the gaze point and the target stimulus was 50 to 1050 ms. The gaze point acted as an extrinsic alerting stimulus that served as an alerting cue, i.e., an alerting signal cue. Alerting affects changes in the internal state and involves the detection of and preparation for a response to an expected signal (Petersen and Posner, 2012), thus, when cued by an alerting signal, subjects respond more quickly when they have a simple response compared to no alerting signal (Fan et al., 2005). During experiments, although alerting signals usually provide little or no information about when the target occurs, they confer a behavioral advantage relative to what would occur in the absence of an alerting signal, thus, subjects with higher alerting efficiency are better able to use alerting signals to increase attention to information (Weinbach and Henik, 2011), prompting the organism to respond to incoming perceptual stimuli in a timely manner (Wang et al., 2016). This also verifies previous research that has shown that alerting efficiency has a certain impact on spatial perceptual responses, and that alerting efficiency affects individual perceptual responses and processing speed (Droit-Volet et al., 2013). To some extent, this suggests that athletes with high alerting efficiency have faster spatial perceptual responses.

According to the direct perception theory, individuals acquire information about the surrounding environment directly from the external world through continuous exploration, which leads to a 'bottom-up' perceptual process. Attentional factors play a crucial role in shaping spatial perception (Vanlessen et al., 2013). Specifically, attention facilitates the processing of visual information, leading to a quicker and more accurate perception of attended stimuli. Alerting is a specific type of attention, and individuals with higher alerting efficiency always maintain relatively high arousal to external stimuli, using alerting signals to increase attention to target information stimuli (Weinbach and Henik, 2011). Meanwhile, attention to the target task accelerates the processing of the relevant visual information stimuli. This partially explains the results that youth athletes with higher alerting efficiency had a higher accuracy rate in the spatial position judgment.

Moreover, we found a significant interaction between emotions and the spatial location in spatial perceptual response time, with youth athletes responding more quickly to targets presented in the lower visual field in the angry than in the happy mood, reaching significant levels in the left lower visual field. Independent evidence suggests that emotional assessments and responses require vertical and horizontal spatial mapping (Schnall, 2014). Some research suggests a pattern of interaction between vertical space and emotion assessment (Lynott and Coventry, 2014). Regarding the representation of affective states in vertical spatial terms ("up = good"/"bad = bottom" metaphors), it has been shown that a position of metaphorical congruence favors the processing of both positive and negative stimuli (Marmolejo-Ramos et al., 2014). Faster processing of angry stimuli was found in the lower position of the visual field (Lynott and Coventry, 2014). Previous studies have shown that positive/negative stimuli facilitate the detection of targets in the upper/lower visual field, even when participants are not explicitly instructed to attend to the stimulus valence. This suggests that the emotion-spatial link is not influenced by the type of the stimulus, but rather by the emotional state, with a greater effect on the vertical than on the horizontal axis (Damjanovic and Santiago, 2016). Amorim and Pinheiro (2019) found a significant effect under negative emotion conditions, with faster responses to targets presented in the lower/left than in the upper/right position. Therefore, the results of this study partially suggest that the emotion-space association on the vertical axis appears to be more automatic. Meanwhile, a main effect of the spatial location was observed in spatial position judgment accuracy, with post-hoc analysis indicating that youth athletes had the highest accuracy in judging targets presented in the upper-left location and the lowest accuracy in the lower-left visual field. Combining event-related potential research, we speculate that this result may be due to the different perceived spatial locations, resulting in different amounts of mental energy being used (Vogel and Luck, 2000).

More importantly, we found a significant interaction between alerting efficiency and emotions in spatial location judgment accuracy, with the high alerting efficiency group having lower spatial location accuracy in the angry state than in the happy state, and higher spatial location accuracy in the happy state compared to the low alerting efficiency group, which may be related to the moderating effect of emotion on alerted attention. Some studies have found that emotions influence attention mainly by affecting the alerting network of attention (Cohen et al., 2011; O’Toole et al., 2011). On the one hand, negative emotions, especially angry emotion conditions, can induce the greatest alerting effect (Techer et al., 2015), as demonstrated by the fact that alerting was most effective in negative emotions, i.e., individuals presented reduced cognitive flexibility and accuracy in negative affective states (Compton et al., 2004; Moriya and Tanno, 2009). Additionally, it has been found that task difficulty varies in its effect on attentional networks, with negative emotional stimuli promoting increased alerting efficiency in the attentional system in tasks with low cognitive loads (Cohen et al., 2011; O’Toole et al., 2011). The spatial perceptual selection task used in this study was not very difficult and can be classified as a low-load task. The results of this study show that even in a low-load task, negative emotions can interfere with the accuracy of spatial location perception, so that the accuracy of spatial location judgment of athletes with high alerting efficiency, yet being in an angry mood, was significantly lower than in a happy mood. On the other hand, it may be related to the moderating effect of positive emotions on alerting. Some research has found that positive emotions have a positive effect on the alerting dimension of the attentional network (Levasseur et al., 2022).

In conclusion, these results support our hypothesis. Firstly, youth athletes with high alerting efficiency showed some spatial perceptual response advantage over those with low alerting efficiency. It can be inferred from our results that the faster the spatial response, the higher the accuracy of individuals with high alerting efficiency. This means that athletes with high alerting efficiency can make more accurate decisions based on the changes of their opponent and his/her spatial position on the field in a shorter period of time, which suggests that alerting efficiency is an important factor affecting athletes' competitive performance. Importantly, some research has shown that acute exercise can improve the function of the individual's alerting network (Huertas et al., 2011).

Furthermore, our results support the notion that spatial perception of youth athletes with high alerting efficiency is more affected by emotions than in athletes with low alerting efficiency. Therefore, for athletes, especially those with high alerting efficiency and in an angry emotional state in the competition situation, timely and effective use of emotion regulation strategies (e.g., cognitive reappraisal) can result in a better use of their own spatial perceptual response advantages. Also coaches should pay more attention to the psychological state of athletes in the specific situation during daily training and/or competition, and adopt appropriate emotional regulation strategies considering athletes' own characteristics. Developing training programs to guide athletes to actively adopt strategies to regulate their emotions is recommended, so that athletes always maintain a good emotional state and thus, sports performance. This study provides a reference for training of athletes' spatial perception and professional perceptual ability and enriches the index system of mental selection for youth athletes.

## Limitations and Future Research

Our study is not without some limitations, which should be addressed in future studies. Firstly, there was a lack of objective physiological monitoring metrics in this study. Future research should consider some indicators of people's emotional responses such as skin conductance, heart rate variability, facial muscles, and other non-invasive measurements. Additionally, the ANT study can benefit from the use of event-related functional magnetic resonance imaging to explore brain regions related to the attention system (Fan et al., 2005). Therefore, further research is necessary to use these methods to explore the influences on spatial perception in athletes and the neural mechanisms that interact with event-related potential (ERP) and functional magnetic resonance imaging (fMRI). Moreover, it would be beneficial to refine the research paradigm by incorporating sample characteristics and integrating it with the movement context to enhance the ecological validity of experimental studies.

Secondly, we did not control for moderating variables or possible confounders. Previous studies have hypothesized that people's desires and preferences influence their processing of visual stimuli, thereby directing the visual system to favor what is consciously presented. The motivation level influences spatial perception, and thus can be considered for inclusion in future studies. Additionally, in studies where no specific physiological assessment was performed, the menstrual status of the female athlete can be indicated at the time of measurement, because for female athletes, the different phases of the menstrual cycle directly affect physiological variables such as the heart rate, the emotional state, and body temperature. Furthermore, although we claim that only healthy youth athletes participated in this study, this was self-reported by athletes, and we did not adequately assess and measure the quality of sleep of participants. Future studies should include sleep quality as a covariate to ensure that these potential confounding variables do not undermine the internal validity of the study. Meanwhile, in future research we should also consider emotional intelligence of athletes as well as previous training they have received in mental skills. One study found a moderate positive correlation between players' perception of emotions and their ability to concentrate (González-Hernández et al., 2023). There is also a positive correlation between athletes' ability to self-regulate their emotions and their levels of self-confidence and achievement motivation, which can improve their ability to cope in sport, which can further influence their athletic performance (Jooste et al., 2023).

Thirdly, our intervention for neutral emotional states should be further developed and adapted to a relevant sample of athletes. Given the complex interactions between alerting attention and emotions in previous studies, inconsistent results of increases and decreases were demonstrated. Therefore, the design of this study included two emotion groups (happy and angry), yet it lacked a control group that did not receive any emotion induction. Inclusion of a control group would help better compare and explain the effects of emotions on spatial perception. In future research, we intend to recruit participants to address this limitation and enhance the comprehensiveness of our findings.

## Conclusions

In conclusion, the results of this study show that the Attention Network Test has good validity in the youth athlete population; youth athletes with high alerting efficiency have fast spatial perceptual responses and high accuracy, and the accuracy of the spatial position judgment is more easily influenced by emotions. These findings suggest that youth athletes with high alerting efficiency show a superior spatial perceptual response compared to those with low alerting efficiency.

As we all know, in highly competitive sports where athletes are required to make quick and accurate decisions based on constantly changing environmental information, individual perceptual reaction times are often less than 1 s. Our findings indicate that the average spatial reaction time of athletes with high alertness was approximately 8 s less than that of athletes with low alertness, as well as it was by 0.3% more accurate, which is certainly an overwhelming advantage for competitive sports. Notably, in a study of the effects of three different activity conditions on three attentional functions, acute exercise was found to have a beneficial effect on the alerting network function (Huertas et al., 2011). Considering that youth athletes with high alerting efficiency showed a superior spatial perceptual response advantage, with faster responses (8 s less) and higher accuracy (0.3%), this potential intervention could be used in future studies to develop appropriate exercise interventions to improve athletes' alerting efficiency and further enhance their performance.

In summary, alerting efficiency can be used as one of the objective indicators to evaluate and diagnose the spatial perceptual ability of youth athletes, providing scientific reference and empirical basis for psychological selection and specialized perceptual training of youth athletes.
